# Trimethylamine N-Oxide and Impaired Spermatogenesis in the Gut–Testis Axis: A Focused Review of Current Evidence

**DOI:** 10.3390/biology15131078

**Published:** 2026-07-06

**Authors:** Xinyang Zhang, Jialin Luo, Xiang Zhang, Fang Yang, Xiaojin Zhang, Xujun Yu, Liang Dong

**Affiliations:** 1 School of Medical and Life Sciences, Chengdu University of Traditional Chinese Medicine, Chengdu 611137, China; xyangzhang2026@163.com (X.Z.); lu0o0511@163.com (J.L.); oxoiang828@163.com (X.Z.); yuxujun@cdutcm.edu.cn (X.Y.); 2 School of Health Preservation and Rehabilitation, Chengdu University of Traditional Chinese Medicine, Chengdu 611137, China; yangfang@cdutcm.edu.cn; 3 Library, Chengdu University of Traditional Chinese Medicine, Chengdu 611137, China; zhangxiaojin@cdutcm.edu.cn

**Keywords:** trimethylamine N-oxide, spermatogenesis, asthenozoospermia, gut–testis axis, gut microbiota, Leydig cells, oxidative stress

## Abstract

Male infertility refers to the inability of a male partner to contribute to pregnancy after 12 months or more of regular unprotected intercourse. It is frequently associated with impaired spermatogenesis, and many cases cannot be completely explained by genetic, inflammatory, endocrine, or environmental factors. Trimethylamine N-oxide (TMAO) is generated when gut bacteria convert dietary choline, L-carnitine, and betaine into trimethylamine (TMA), which is oxidized in the liver mainly by flavin-containing monooxygenase 3 (FMO3). This focused review evaluates current evidence on TMAO and impaired spermatogenesis within the gut–testis axis. The rationale is biologically plausible because sperm motility depends on coordinated glycolytic and mitochondrial energy metabolism, whereas Leydig cell steroidogenesis depends on mitochondrial cholesterol transport and redox balance. Human observational studies associate TMAO with asthenozoospermia and Leydig cell-related markers, particularly insulin-like peptide 3 (INSL3), while mouse studies suggest testicular injury and reduced spermatogenesis after TMAO-related exposure. The Hippo/Yes-associated protein (YAP)–mitochondria–steroidogenic acute regulatory protein (StAR) axis has been proposed as one possible mechanism, but direct reproductive tract exposure, blood–testis barrier kinetics, and human validation remain unresolved. Overall, TMAO should be considered as a candidate metabolic mediator and not a proven causal factor or therapeutic target in male infertility.

## Simple Summary

Male infertility cannot always be fully explained by genetic defects, infection, varicocele, endocrine disorders, or other established clinical factors. For unexplained or multifactorial cases of male infertility, the gut microbiota and its small-molecule metabolites may provide an additional perspective for interpretation. Trimethylamine N-oxide (TMAO) is a circulating molecule regulated by diet, gut production of trimethylamine (TMA), hepatic metabolism, and renal clearance. Elevated trimethylamine N-oxide may be associated with impaired spermatogenesis. a process highly dependent on coordinated energy metabolism and testicular cell-to-cell support. Human observational studies have linked TMAO with decreased sperm motility and Leydig cell-related hormonal biomarkers, while mouse studies suggest that TMAO exposure may contribute to testicular injury and reduced spermatogenesis. However, current human data remain insufficient to confirm a causal relationship. This focused review summarizes the available evidence, separates human findings from animal and indirect mechanistic data, and provides a critical assessment of what remains uncertain. A clearer understanding of this topic may help guide future studies on metabolic and lifestyle factors related to male reproductive health.

## 1. Introduction

Infertility is a major reproductive health concern worldwide. According to the 2023 World Health Organization report, approximately one in six adults experiences infertility during their reproductive lifetime [1]. Within this overall burden, male factors play a significant role in infertility, and male-factor infertility is commonly associated with abnormal semen parameters, including oligozoospermia (low sperm concentration or count), asthenozoospermia (reduced sperm motility), teratozoospermia (abnormal sperm morphology), or mixed semen abnormalities involving more than one semen parameter [2,3]. These semen abnormalities can reduce the likelihood of natural conception and may also adversely affect assisted reproduction outcomes. The potential for a decrease in sperm counts also has public health implications, as evidenced by recent trends in decreasing sperm counts [4].

A relatively stable testicular microenvironment is required for spermatogenesis. This microenvironment is maintained by the germ cells, Sertoli cells, Leydig cells, the blood–testis barrier and the local immune environment and is essential for the process of spermatogenesis. Oxidative stress and mitochondrial dysfunction are important mechanisms discussed in male infertility. Oxidative stress is defined as an imbalance between reactive oxygen species (ROS) production and antioxidant defense [5,6]. The accumulation of ROS can cause injury to sperm membrane lipids, mitochondria, proteins, and deoxyribonucleic acid (DNA), which can lead to a decrease in sperm motility and the ability of sperm to fertilize eggs [5,6]. Mitochondrial cholesterol transport and cellular energy status are also important in testosterone production in the Leydig cell [7,8]. The steroidogenic acute regulatory protein (StAR) is an important protein for the delivery of cholesterol to mitochondria [7,8]. Hence, metabolic factors of the system, which affect mitochondrial function, redox balance, or steroidogenesis, may also influence spermatogenesis.

Beyond its structural organization, the testis is also metabolically specialized. Sperm motility depends on adenosine triphosphate (ATP) generated through coordinated glycolytic and mitochondrial energy metabolism, and both pathways are vulnerable to oxidative stress and mitochondrial injury. Leydig cell steroidogenesis depends on mitochondrial cholesterol transport, redox balance, and adequate cellular energy status. Sertoli cells further support developing germ cells by regulating nutrient availability and metabolic substrate supply, including lactate production. Therefore, circulating xenobiotics or gut-derived metabolites may affect male reproductive function not only by injuring isolated testicular cell types, but also by perturbing energy metabolism, mitochondrial homeostasis, and intercellular metabolic cooperation within the testicular microenvironment.

The gut microbiota has been increasingly recognized to have a role in regulating distant organs in recent years. The bacterial community of the gut has the potential to affect the homeostasis of the host via metabolites, immune signaling, inflammatory responses, and endocrine regulation [9]. The gut–testis axis has been conceptualized in male reproductive research to suggest that changes in gut microbiota can impact the testicular microenvironment via systemic mechanisms [10,11]. It is still however a developing field and many studies are still largely associational in nature. Therefore, a shift in the gut microbiome should not be considered to be an individual causal factor of male infertility.

**Figure 1:**
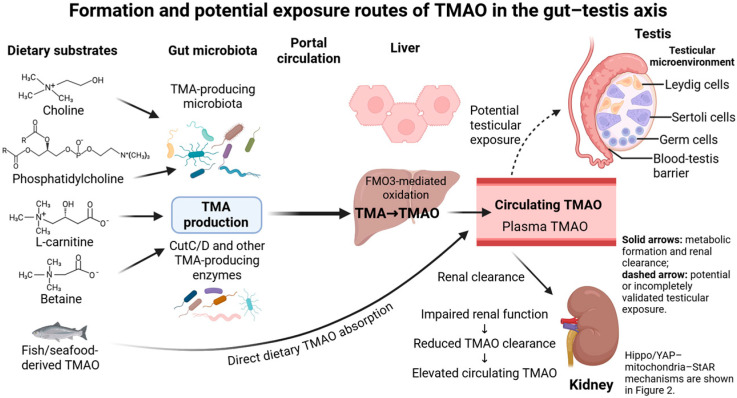
Formation and potential exposure routes of trimethylamine Noxide (TMAO) in the guttestis axis. Dietary precursors, including choline, phosphatidylcholine, Lcarnitine, and betaine, can be converted by gut microbial enzymes into trimethylamine (TMA). Microbial TMA production may involve choline trimethylamine-lyase-related enzymes, including Choline trimethylamine-lyase system (*CutC*/*D*). TMA enters the portal circulation and is oxidized mainly in the liver by flavin-containing monooxygenase 3 (FMO3) to form circulating TMAO. Fish and seafood may also contribute preformed dietary TMAO. Circulating TMAO levels depend on microbial TMA production, hepatic oxidation, direct dietary intake, and renal clearance. Solid arrows show metabolic formation and clearance pathways; the dashed arrow denotes potential testicular exposure that remains incompletely validated. The bloodtestis barrier (BTB) is shown as part of the testicular microenvironment, and the possible Hippo/Yesassociated protein (YAP)mitochondria–steroidogenic acute regulatory protein (StAR) mechanism is detailed in Figure 2.

Metabolites from gut microbiota have a relatively well-defined metabolic origin, such as trimethylamine N-oxide (TMAO). Gut bacteria can break down nutrients like choline, L-carnitine, and betaine and convert them into trimethylamine (TMA). TMA then travels to the liver via portal circulation, where it is oxidized to TMAO by flavin-containing monooxygenases, specifically flavin-containing monooxygenase 3 (FMO3) [12,13,14], as shown in Figure 1. Studies on TMAO have primarily been conducted in the areas of cardiovascular disease, chronic kidney disease (CKD), and metabolic disorders [15,16,17]. Though these studies do not provide direct evidence that it is implicated in male infertility, they do lend support to the notion that metabolites produced by microbiota can have an impact on distant tissues and organs via the systemic circulation.

**Figure 2:**
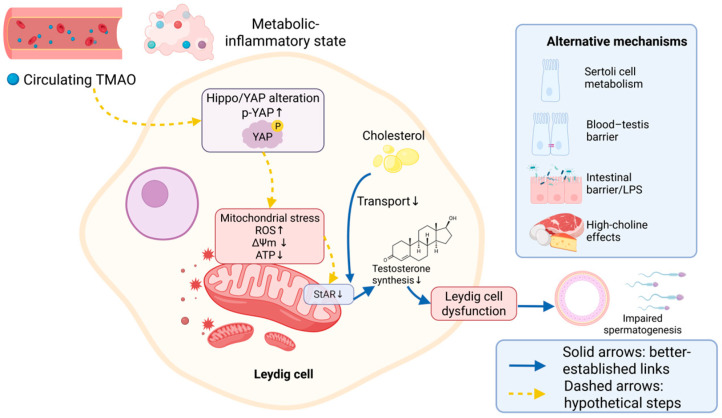
Proposed Hippo/Yes-associated protein (YAP)–mitochondria–steroidogenic acute regulatory protein (StAR) mechanism in Leydig cells. Circulating trimethylamine N-oxide (TMAO), or a related metabolic-inflammatory state, may be associated with altered Hippo/YAP signaling, increased YAP phosphorylation, mitochondrial stress, increased reactive oxygen species (ROS), reduced adenosine triphosphate (ATP) production, decreased StAR expression, and impaired testosterone synthesis. These changes may contribute to Leydig cell dysfunction and impaired spermatogenesis. The figure also shows alternative or parallel mechanisms, including Sertoli cell metabolic disturbance, blood–testis barrier disruption, intestinal barrier/lipopolysaccharide (LPS)-related signaling, and high-choline diet effects. Solid arrows indicate relatively better-supported links from preclinical evidence, whereas dashed arrows indicate hypothetical or indirect steps that require further validation in human reproductive tissues. This pathway should be interpreted as a candidate mechanism rather than a confirmed causal pathway in human testes.

The link between TMAO and poor sperm quality is still weak. In a matched case–control study in a hospital setting, a correlation between raised TMAO and risk of asthenozoospermia was found [18]. The preclinical studies revealed that TMA-producing bacteria and exposure to TMAO were related to mitochondrial damage in the testes, dysfunction of the Leydig cells, and abnormal spermatogenesis in mice [19]. The results are informative, but are not yet confirmed. Most studies that have been conducted in humans are observational, and circulating TMAO levels have been shown to be influenced by diet, consumption of fish and red meat, renal function, obesity, diabetes, medications, and interpopulation differences [16,20,21,22]. Animal and cell-based studies may offer mechanistic insight, but cannot replace human evidence due to differences in species, dose of exposure, experimental conditions, and clinical endpoints [19,23].

This focused review evaluates the current evidence on TMAO and impaired spermatogenesis within the gut–testis axis. Unlike broader reviews on the gut microbiota and male reproductive health, this review separately examines human observational associations, microbial sources of TMA, preclinical evidence, indirect mechanistic studies, and discusses the strengths and limitations of each type of evidence. It also emphasizes that circulating TMAO levels are shaped by diet, gut microbiota composition, hepatic FMO3 activity, and renal clearance [12,13,16,20]. In addition, this review considers the Hippo/Yes-associated protein (YAP)–mitochondria–StAR axis as provisional evidence, rather than a causal pathway already confirmed in human testes [19]. Overall, this review is not to present TMAO as a confirmed cause of male infertility or a validated therapeutic target, but to clarify what the current literature supports, what remains uncertain, and what types of studies are needed next.

## 2. Literature Search and Evidence Selection

This article is a focused narrative review, not a systematic review, scoping review, or meta-analysis. Therefore, no quantitative synthesis was performed, and no Preferred Reporting Items for Systematic Reviews and Meta-Analyses (PRISMA) flow diagram was used. This section is included to improve the transparency of this focused narrative review by describing the literature search, review scope, and evidence selection strategy. It is not intended to present experimental materials or methods. Rather, it explains how key human, microbiome, animal, cellular, and mechanistic studies were identified and organized to evaluate the possible relationship between trimethylamine N-oxide (TMAO), gut microbial metabolism, and male reproductive dysfunction.

The literature was searched mainly in PubMed, Web of Science, Google Scholar, and relevant journal websites, with searches last updated in June 2026. Search terms included “trimethylamine N-oxide”, “TMAO”, “trimethylamine”, “spermatogenesis”, “asthenozoospermia”, “male infertility”, “gut microbiota”, “gut–testis axis”, “Leydig cell”, “Sertoli cell”, “blood–testis barrier”, “StAR”, “Hippo signaling”, “YAP”, “mitochondria”, “oxidative stress”, “choline”, “carnitine”, and “dietary red meat”. Reference lists of key articles and related reviews were also checked manually to reduce the risk of missing relevant studies.

Studies reporting reproductive outcomes directly in males were given priority. The outcomes were semen parameters, sperm motility, total motile sperm count, sperm morphology, testicular histology, testosterone levels, Leydig cell function, Sertoli cell function, blood–testis barrier integrity, and spermatogenic dysfunction. Aside from studies specifically related to male reproduction, a few studies from the cardiovascular, renal, metabolic, and immunoinflammatory fields were also included. These studies were primarily employed to illuminate the biology, potential pathways, and potential confounds of TMAO, such as dietary factors, fish and red meat consumption, gut microbes, FMO3 activity, and renal clearance. They were not necessarily used as an actual trace for a function of TMAO with regard to male infertility.

Evidence was evaluated with consideration of study design, relevance of the study population to male reproductive outcomes, adjustment for key confounders, inclusion of microbiome or metabolite data, availability of animal or cellular mechanistic evidence, and reporting of clinically relevant reproductive endpoints. These endpoints included semen parameters, hormone levels, pregnancy rate, assisted reproduction outcomes, and live birth rate. On this basis, evidence was organized into five categories: human association, microbial source, preclinical experimental, indirect mechanistic, and potential intervention evidence. The value and limitations of each are discussed below.

## 3. Evidence Linking TMAO to Impaired Spermatogenesis

### 3.1. Human Association Evidence

Human evidence linking TMAO to impaired spermatogenesis remains limited. In 2025, Xing et al. [18] published a hospital-based, matched case–control study in China. The study included 314 pairs of asthenozoospermia cases and normozoospermic controls, matched by age, body mass index (BMI), and smoking status [18]. The investigators classified cases and controls according to semen parameters and measured plasma TMAO and several related metabolites, including choline, betaine, L-carnitine, methionine, and dimethylglycine. Compared with the lowest quartile, the highest quartile of plasma TMAO was associated with higher odds of asthenozoospermia (odds ratio (OR) = 1.80; 95% confidence interval (CI): 1.16–2.81). When analyzed per one-standard-deviation increase, TMAO was also associated with higher odds of asthenozoospermia (OR = 1.31; 95% CI: 1.12–1.55) [18]. These results suggest that TMAO may be a candidate metabolite for further study. The value of this study lies in its direct analysis of circulating TMAO in relation to human semen phenotypes, rather than depending solely on animal experiments or evidence from cardiovascular and metabolic research. However, asthenozoospermia is a multifactorial disorder. Oxidative stress, reduced mitochondrial membrane potential, sperm DNA fragmentation, varicocele, infection and inflammation, metabolic abnormalities, and lifestyle factors may all affect sperm motility [2,5,6]. Therefore, the association observed in this study should not be directly interpreted as evidence that TMAO causes asthenozoospermia.

Several limitations of the Xing et al. study should also be noted. First, the case–control design compared TMAO levels and semen phenotypes at a single time point and could not determine whether elevated TMAO preceded asthenozoospermia or occurred after reproductive dysfunction or lifestyle changes. Second, although renal insufficiency was excluded to minimize confounding from renal clearance, residual confounding from diet (particularly red meat and fish intake), smoking, antibiotic use, obesity, and diabetes may persist. Third, a single plasma TMAO measurement captures only a snapshot of metabolic status and cannot reflect long-term exposure. Fourth, the hospital-based population may not be generalizable, and findings require replication in larger, population-based cohorts across diverse regions.

A further limitation is that circulating plasma TMAO should not be interpreted as a direct mirror of local exposure within the male reproductive tract. Spermatogenesis occurs in a specialized seminiferous microenvironment that is partly protected by the blood–testis barrier (BTB), which is formed mainly by junctional complexes between Sertoli cells. Whether TMAO can enter the adluminal compartment of the seminiferous epithelium through passive diffusion, transporter-mediated movement, or other routes remains unknown. In addition, it is unclear whether TMAO itself, or a related metabolic-inflammatory state associated with elevated TMAO, can alter BTB integrity or Sertoli cell junctional homeostasis. Therefore, the association between plasma TMAO and semen phenotypes should be interpreted cautiously until paired plasma, seminal plasma, or testicular tissue measurements are available.

A related gap is the lack of paired plasma and seminal plasma metabolomic data. Seminal plasma is more directly connected to the male reproductive tract than peripheral blood and may help determine whether circulating TMAO reflects local reproductive exposure. Untargeted seminal plasma metabolomics and lipidomics have increasingly been used to characterize metabolic signatures associated with male infertility [24,25,26]. However, current TMAO-related studies have not established whether TMAO is consistently detectable in human seminal plasma or whether seminal TMAO correlates with sperm motility, sperm membrane lipid composition, sperm DNA integrity, or reproductive outcomes. Therefore, future studies should measure TMAO and related metabolites in paired plasma and seminal plasma samples, together with semen parameters and reproductive hormones, before plasma TMAO can be interpreted as a marker of local gut–testis axis activity.

Besides the association with asthenozoospermia, Ayvaci et al. found that serum TMAO was related to Leydig cell-related markers in cryptorchid boys [27]. This study concluded that the serum TMAO level was raised in cryptorchidism boys and the serum insulin-like peptide 3 (INSL3) level was decreased. INSL3 is a small, peptide hormone that is secreted by the mature Leydig cell and the relative stability of its expression and secretion can be used as a reflection of Leydig cell differentiation status and cell number [28]. The result further suggests human evidence for the association of TMAO with testicular endocrine phenotypes. Yet, the shortcomings of this study are also obvious. It was not adult men with infertility who were the participants, but rather children with cryptorchidism. There was no evaluation of the gut microbiota or of functional genes related to microbial TMA production, and hence no conclusion could be drawn about whether the increase in TMAO was due to altered gut microbial metabolism. Therefore, this study should be considered suggestive evidence and not as proof that TMAO plays a role in the pathophysiology of impaired spermatogenesis in adult men.

### 3.2. Microbial Source Evidence

All the above suggest the metabolic basis that the gut microbiota should be able to produce TMA for the formation of TMAO. The question is whether abnormal semen parameters correlate with alterations in the number and/or function of TMA-producing bacteria or with the number of functional genes encoding TMA production in men with abnormalities. Wang et al. examined the metagenomes in feces from 107 subjects and serum TMAO levels and semen parameters were also measured. It was found that some of the taxa associated with the choline-to-TMA ratio, such as *Phocaeicola massiliensis*, *Veillonella* spp., and *Klebsiella pneumoniae*, were relatively enriched in men with abnormal semen parameters. Additionally, serum TMAO levels were also negatively associated with semen volume and total sperm count, and motile sperm count [19]. These findings indicate that TMA/TMAO pathway could represent not only the changes in circulating metabolites, but also the gut microbial function.

Microbial source evidence is most useful in providing a possible explanation of the origin of the signal. Then, enrichment of TMA-producing bacteria or the functional genes in men with abnormal semen parameters can mean that the high level of TMAO is not just a plasma finding, but rather that gut microbial function is altered [19,29]. But the abundance of the microbes as a proportion of the whole does not necessarily reflect metabolic flux. As the amount of TMA-producing bacteria increases, it does not necessarily result in an increase in the amount of TMAO. The final plasma TMAO level depends on the availability of substrates, microbial interactions, TMA absorption, FMO3 activity, and renal clearance [16,20,21,30]. Future research should separate the findings of observational studies in humans from validation studies based on animals. The former tend to be more directive, while the latter are more like a mechanistic inference. To clarify the microbial source of the TMA/TMAO pathway, representative human gut bacteria and associated functional genes implicated in microbial TMA production are summarized in Table 1. These taxa influence the TMAO pathway by contributing to intestinal TMA availability, whereas the subsequent oxidation of TMA to TMAO depends mainly on host hepatic FMO3 activity.

### 3.3. Animal Experiments and Preclinical Support

In the study performed by Wang et al., the most comprehensive preclinical evidence to date for a potential role of TMAO in impaired spermatogenesis is given [19]. In addition to one TMAO gavage experiment, patient fecal microbiome analysis, fecal microbiota transplantation into germ-free mice, a high-choline diet, and colonization by TMA-producing strains, direct exposure to TMAO was performed. This enabled the authors to investigate the TMAO pathway at different levels, starting from the microbiota upstream, the substrates in the food, specific strains of bacteria, to the final metabolite, to determine if this pathway was linked to testicular injury.

Methodologically, Wang et al. first compared gut microbial features between men with abnormal semen parameters and controls, focusing on taxa and functional signals linked to TMA production; they then transplanted patient-derived fecal microbiota into germ-free mice to assess effects on testicular tissue, sperm morphology, and androgen synthesis. The study also used a high-choline diet to increase endogenous TMAO production, colonization with TMA-producing strains to identify candidate bacterial sources, and oral TMAO administration to test the effect of the terminal metabolite itself [19].

The outcome measures included testicular histological changes, abnormal sperm morphology, testosterone synthesis-related indicators, mitochondrial membrane potential, ROS, adenosine triphosphate (ATP), Hippo/YAP signaling, and StAR expression [19]. These measures allowed the study to go beyond sperm or testicular morphology. They also involved Leydig cell stress, mitochondrial function, and steroidogenesis.

The results showed that mice receiving microbiota from men with abnormal semen parameters developed abnormal sperm morphology, testicular tissue injury, and reduced androgen synthesis. A high-choline diet and direct TMAO exposure also reproduced part of the injury phenotype. At the same time, Leydig cells showed impaired mitochondrial function, increased ROS, reduced ATP production, and inhibited StAR expression and testosterone synthesis [19]. Single-strain colonization experiments further suggested that some TMA-producing bacteria may be important components of this pathway. Compared with the human study by Xing et al., the Wang et al. study examined microbiota, metabolites, and tissue injury within the same experimental framework, providing a more complete evidence chain, although it remains preclinical.

The Wang et al. study also has several limitations. First, the immune system, gut development, and metabolic status of germ-free mice differ from those of conventional mice and humans. Therefore, fecal microbiota transplantation in germ-free mice may be influenced by the distinct physiological state of these animals. Second, fecal microbiota transplantation transfers not only TMA-producing bacteria but also many other bacteria, metabolites, and immune stimuli, so observed phenotypes cannot be exclusively attributed to TMAO. Third, a high-choline diet elevates TMAO but may also alter one-carbon metabolism, phospholipid metabolism, and other choline-related pathways. Fourth, the dose, duration, and route of TMAO gavage do not fully match long-term dietary exposure in humans and may amplify acute injury signals. Fifth, spermatogenic cycles, gut microbial structures, FMO3 activity, and testicular stress responses differ between mice and humans. Therefore, this study is better viewed as mechanistic support rather than a clinical conclusion. It also did not report clinically relevant reproductive endpoints, such as natural pregnancy rate or live birth rate, and lacked validation in human testicular samples. Further validation in human cohorts and clinically relevant samples is still needed.

### 3.4. Indirect Support and Research Gaps

Broader literature searches have shown that several non-reproductive studies have linked TMAO with inflammation, oxidative stress, and dietary patterns. In an aged rat model, Li et al. found that plasma TMAO levels were higher in aged animals and were accompanied by impaired endothelium-dependent relaxation, increased inflammatory factor expression, and increased superoxide production. After reducing TMA production with 3,3-dimethyl-1-butanol (DMB), TMAO levels and some vascular injury markers improved [31]. This study did not examine the testis or sperm. However, it suggests that TMAO may be related to oxidative stress and inflammatory responses, both of which are frequently discussed in studies of male infertility and sperm dysfunction [5,6,31].

Another useful line of evidence comes from dietary intervention research. Wang et al. studied the comparison of red meat, white meat, and non-meat protein diets in healthy adults. They determined that a red meat diet led to the generation of TMA precursors, an increased formation of TMA/TMAO from carnitine, and reduced renal excretion of TMAO. Plasma levels of TMAO were reduced within weeks after quitting the red meat diet [21]. These results indicate that systemic TMAO levels can vary substantially according to dietary intake and renal excretory function. This variability is important when interpreting possible reproductive associations, because circulating TMAO may reflect a broader dietary, renal, and metabolic context rather than a testis-specific exposure. Systemic TMAO-related changes could plausibly influence the testicular microenvironment through circulation-mediated oxidative, inflammatory, or metabolic signaling. However, without information on diet, renal function, and related metabolic factors, a single plasma TMAO measurement should not be used to draw direct conclusions about male reproductive function.

These indirect studies can support the discussion of possible mechanisms and confounding factors, but they should not be considered direct evidence in male reproduction. They help explain why TMAO warrants attention as a candidate systemic metabolic signal and why future studies should evaluate reproductive tract exposure, seminal plasma metabolomics, testicular tissue responses, and clinically relevant fertility endpoints. The role of TMAO in impaired human spermatogenesis therefore remains to be examined in specific population cohorts and intervention studies.

### 3.5. Synthesis of Evidence

Taken together, the available evidence suggests a preliminary evidence chain between TMAO and impaired spermatogenesis. Human studies provide observational associations between TMAO and asthenozoospermia or testis-related endocrine phenotypes [18,27]. Microbial source evidence suggests that the upstream TMA-producing pathway may be linked to changes in gut microbial function [19,29]. Animal experiments using fecal microbiota transplantation, single-strain colonization, high-choline diet, and direct TMAO exposure provide preclinical support for testicular injury and abnormal spermatogenesis [19]. Non-reproductive studies further suggest that TMAO is related to oxidative stress, inflammatory responses, dietary patterns, and renal excretion [21,31]. These lines of evidence are directionally connected, but they do not have the same evidentiary strength. Key characteristics of the included studies and their main findings are summarized in Table 2.

## 4. Candidate Mechanisms from Preclinical Studies

### 4.1. Mitochondrial Stress and Steroidogenesis

Mitochondria are important for both spermatogenesis and androgen synthesis in Leydig cells. In sperm, mitochondria provide adenosine triphosphate (ATP) for motility and are closely linked to oxidative stress through reactive oxygen species (ROS) production. Mitochondrial dysfunction can increase electron leakage from the respiratory chain and promote ROS generation. When ROS production exceeds antioxidant defense capacity, oxidative stress occurs. Excessive ROS can then damage mitochondrial membranes, mitochondrial proteins, membrane lipids, and deoxyribonucleic acid (DNA), reduce mitochondrial membrane potential, and further impair ATP production. This reciprocal relationship may amplify mitochondrial stress and oxidative injury, thereby reducing sperm motility and fertilizing capacity [6,32,33]. The mitochondria are also involved in the initial stages of the formation of testosterone in Leydig cells. One of the first steps in the biosynthesis of steroids is for cholesterol to be internalized into mitochondria and then metabolized to pregnenolone in the mitochondrial compartment. StAR is mainly involved in cholesterol transport into mitochondria [7,8]. Thus, a circulating metabolite that influences the activity of the mitochondria, redox status, or StAR expression potentially could have indirect impacts on testosterone production in Leydig cells and on spermatogenesis.

The decrease in mitochondrial membrane potential and ATP production observed in preclinical TMAO-related models suggests mitochondrial bioenergetic stress in testicular cells [19]. Because mitochondrial oxidative phosphorylation (OXPHOS) supports ATP generation, impaired OXPHOS may reduce electron transport efficiency, increase electron leakage, and promote mitochondrial reactive oxygen species (ROS) generation in sperm and testicular cells [6,32,33]. Mitochondrial membrane dynamics, including fusion, fission, cristae remodeling, and organelle contact sites, are important for maintaining mitochondrial structure, quality control, and bioenergetic adaptation [34]. Different mitochondrial fission patterns may be associated with mitochondrial degradation or biogenesis, suggesting that fission is not only a morphological endpoint but also part of mitochondrial quality control [35]. Therefore, disturbance of fusion-related regulators, such as mitofusins and optic atrophy 1 (OPA1), or fission-related regulators, such as dynamin-related protein 1 (DRP1), could theoretically amplify mitochondrial fragmentation, ATP deficiency, and oxidative injury in testicular cells. However, these mitochondrial dynamic changes have not been directly observed in testicular cells following TMAO exposure. This framework remains hypothesis-generating and should not be interpreted as a confirmed mechanism.

### 4.2. Hippo/YAP Signal as a Possible Regulatory Link

Wang et al. found that after exposure to TMAO, mouse testes and Leydig cells exhibited changes in Hippo signaling, increased YAP phosphorylation, reduced mitochondrial membrane potential, elevated ROS, impaired ATP production, as well as reduced StAR expression and lower testosterone synthesis [19]. These findings show that the exposure to TMAO is correlated with Hippo/YAP signaling and with the damage to mitochondria and steroidogenesis in Leydig cells. They now provide the primary mechanistic clues to the possible effect of TMAO on spermatogenesis: raising the dietary substrates or modifying TMA-producing microbial function may lead to higher levels of circulating TMAO. Once the TMAO and/or the metabolic and inflammatory state that it represents have reached the testicular microenvironment, they can potentially influence Hippo/YAP signaling. It can also initially raise mitochondrial ROS levels and lower the energy metabolic efficiency. The following step may be the damage to mitochondria, which in turn may affect the process of cholesterol transport by StAR and the synthesis of testosterone, which results in Leydig cell dysfunction and consequently in impaired spermatogenesis [19,31,36].

But it should be regarded as a possible mechanistic link rather than a confirmed pathway. The study noted that after exposure to TMAO, there were simultaneous changes in multiple indicators, and temporal sequence and direct causal relationships among the changes have yet to be completely explained. The question is whether TMAO directly impacts Hippo/YAP signaling, whether Hippo/YAP changes always precede the mitochondrial damage and whether StAR downregulation is solely dependent on this pathway. These questions need to be further validated. Importantly, Leydig cell dysfunction should not be interpreted as an isolated cell-autonomous event, because spermatogenesis depends on coordinated endocrine, paracrine, and metabolic communication among Leydig cells, Sertoli cells, and developing germ cells.

### 4.3. Leydig–Sertoli–Germ Cell Metabolic Cooperation

The proposed Hippo/YAP–mitochondria–StAR pathway should be interpreted within the broader metabolic organization of the seminiferous epithelium. Leydig cells provide testosterone, which is required for normal Sertoli cell function and spermatogenesis [37]. Sertoli cells, in turn, support developing germ cells by regulating nutrient availability, maintaining blood–testis barrier integrity, and supplying metabolic substrates, including lactate [38,39,40]. Therefore, reduced testosterone synthesis or mitochondrial stress in Leydig cells may secondarily affect Sertoli cell support and germ cell development.

This point is particularly relevant to TMAO-related mechanisms. Wang et al. reported mitochondrial stress, increased reactive oxygen species, reduced adenosine triphosphate production, decreased StAR expression, and impaired testosterone synthesis in Leydig cells after TMAO-related exposure [19]. These findings suggest a Leydig cell-centered mechanism, but they do not exclude broader seminiferous tubule involvement. It is biologically plausible that TMAO-related metabolic or inflammatory stress could also influence Sertoli cell mitochondrial function, lactate output, tight junction homeostasis, or germ cell energy supply. However, these possibilities have not been directly tested in TMAO-related studies. Thus, the Leydig–Sertoli–germ cell axis should be considered a hypothesis-generating framework rather than a confirmed pathway.

### 4.4. Unverified Steps and Alternative Mechanisms

The Hippo/YAP–mitochondria–StAR axis is currently supported mainly by a single study by Wang et al. [19], and independent replication remains limited. The relationship between StAR downregulation and the impairment of steroidogenesis in Leydig cells has been supported by basic studies [8,37,41]. There is also evidence for a role of Yes-associated protein 1 (YAP1) in avian Leydig cells that may involve its involvement in luteinizing hormone (LH)-induced signaling networks related to the production of testosterone [36]. Other evidence suggests that TMAO may be indirectly associated with inflammatory response, mitochondrial ROS, the NLR family pyrin domain containing 3 (NLRP3) inflammasome, and immunometabolic changes [31,42]. While these studies provide biological plausibility for this mechanism, they do not indicate that this pathway has been confirmed in human testes.

Another unverified mechanism concerns endoplasmic reticulum (ER) stress and the integrated stress response. Mitochondrial dysfunction and excessive mitochondrial reactive oxygen species may disturb protein folding, calcium homeostasis, and organelle communication, thereby activating unfolded protein response pathways. Among these pathways, protein kinase RNA-like endoplasmic reticulum kinase (PERK)-mediated phosphorylation of eukaryotic initiation factor 2 alpha (eIF2α) can reduce global protein translation while promoting stress-adaptive transcriptional programs. If the stress is prolonged or unresolved, downstream C/EBP homologous protein (CHOP) signaling may contribute to apoptotic priming [43,44]. This pathway is relevant because ER stress can crosstalk with mitochondrial quality control and cell death signaling. However, activation of the PERK/eIF2α/CHOP axis has not been directly demonstrated in TMAO-exposed testicular cells. Therefore, ER stress should be considered a plausible but currently unvalidated extension of the mitochondrial stress framework.

Other possible explanations should also be taken into account. Firstly, TMAO might not be directly involved in the Hippo/YAP signaling pathway. It may, therefore, initially enhance mitochondrial ROS, cellular stress, or inflammatory signaling and indirectly impact the phosphorylation of YAP [42]. Second, TMAO or its metabolic counterpart could influence Leydig cell energy metabolism, expression of tight junction proteins and blood–testis barrier stability, which in turn could impact germ cell development [38,45]. Third, TMAO can coexist with alterations in intestinal barrier function, lipopolysaccharide (LPS)-related signaling, systemic low-grade inflammation, or other microbial metabolites. Together, these can also affect the microenvironment of the testes and cause injury [10,46]. Fourth, TMAO may be an osmolyte and protein stabilizer itself and its effects could be concentration-dependent, cell type-dependent, and tissue-dependent. So, increasing plasma TMAO is not necessarily correlated with the toxic exposure to the testis [47]. Fifth, high-choline diet models not only raise TMAO concentrations, but may also impact one-carbon metabolism, methyl donor cycling, phosphatidylcholine production, and sperm membrane lipid composition [48,49,50]. So, all testicular phenotypes observed in a high-choline diet should not be attributed simply to TMAO.

## 5. Discussion

### 5.1. Interpretation of the Available Evidence

The available evidence is consistent with a possible relationship between the TMA/TMAO pathway and male reproductive dysfunction, but the strength of this evidence varies across study types. Human studies provide the most directly relevant observations, microbial studies support a possible upstream source of the TMAO signal, and preclinical studies provide mechanistic evidence for testicular injury and Leydig cell dysfunction studies [18,19,21,27,31]. These lines of evidence are directionally aligned, but they should not be treated as equivalent forms of evidence. Association in humans, microbial functional changes, animal injury models, and indirect non-reproductive studies each answer different questions and carry different levels of certainty.

Among the available human data, the matched case–control study by Xing et al. provides the clearest reproductive association by linking higher plasma TMAO with asthenozoospermia [18]. The study by Ayvaci et al. adds a second human observation by associating serum TMAO with Leydig cell-related endocrine markers in boys with cryptorchidism [27,28]. These findings are important because they involve human reproductive or testicular phenotypes. However, both remain observational, and neither can establish temporal order, direct testicular exposure, or causality. The cryptorchidism study also differs from adult male infertility in population and outcome, so it should be interpreted as suggestive rather than confirmatory evidence.

Microbial and preclinical findings strengthen the biological plausibility of this relationship. Wang et al. linked TMA-producing microbial features, serum TMAO, abnormal semen parameters, and testicular injury in experimental models [19]. This study provides the strongest current support for a possible gut microbial–TMAO–testis pathway. Nevertheless, its animal and cellular findings cannot substitute for validation in human reproductive tissues or clinically relevant fertility outcomes. Indirect evidence from vascular, metabolic, and dietary studies further supports links between TMAO, oxidative stress, inflammation, diet, and renal excretion, but these studies do not directly assess spermatogenesis [21,31]. Therefore, the current literature supports TMAO as a candidate metabolic signal within the gut–testis axis, not as a proven causal factor or validated therapeutic target in male infertility.

### 5.2. Evidence Gaps and Causal Inference

Although the available studies provide several connected lines of evidence, the causal pathway from circulating TMAO to impaired spermatogenesis remains incomplete. The first gap concerns temporal order. Current human data are mainly observational, so they cannot determine whether elevated TMAO precedes abnormal semen parameters or instead reflects diet, metabolic status, renal clearance, inflammation, or other factors associated with male reproductive dysfunction [18,27,28].

The second gap concerns tissue relevance. Most human data are based on circulating TMAO, but plasma levels do not necessarily reflect exposure within the male reproductive tract. TMAO concentrations are shaped by gut microbial TMA production, hepatic FMO3 activity, diet, renal clearance, and metabolic status [16,20,21,29,30]. Without measurements in seminal plasma, testicular tissue, or validated reproductive tract markers, it remains difficult to connect systemic TMAO with local testicular injury.

The third gap concerns mechanistic validation. Preclinical studies have connected TMAO-related exposure with Leydig cell stress, mitochondrial dysfunction, StAR downregulation, and impaired spermatogenesis [19]. However, the order of these events has not been fully established, and the Hippo/YAP–mitochondria–StAR pathway has not been independently validated in human samples. The current evidence therefore supports a plausible mechanistic framework, but not a confirmed causal pathway in human male infertility.

### 5.3. Confounders and Alternative Explanations

#### 5.3.1. Systemic Confounders

A major challenge in interpreting TMAO is that it is not a single-source exposure. Circulating TMAO integrates dietary precursor intake, gut microbial TMA-producing capacity, hepatic FMO3 activity, renal clearance, and metabolic status [16,20,21,22,30]. Therefore, an association between plasma TMAO and impaired semen parameters may reflect several overlapping processes rather than a direct testicular effect of TMAO. For example, higher TMAO may indicate differences in diet, renal clearance, gut microbial metabolism, systemic inflammation, or cardiometabolic status. These factors may themselves affect male reproductive function, making it difficult to isolate TMAO as an independent contributor.

#### 5.3.2. Interpretation of Experimental Models

Experimental models also require careful interpretation. A high-choline diet can raise TMAO levels, but choline participates in one-carbon metabolism, methyl donor cycling, phosphatidylcholine synthesis, and sperm membrane lipid composition [48,49,50]. Thus, reproductive changes after high-choline feeding cannot be attributed solely to TMAO. A further TMAO-independent mechanism may involve choline-driven changes in phospholipid metabolism and sperm membrane lipid composition. Choline is a precursor for phosphatidylcholine synthesis, and changes in phospholipid availability may influence membrane remodeling in spermatozoa [48,50]. Because the sperm plasma membrane contains abundant polyunsaturated fatty acids (PUFAs), altered membrane lipid composition may increase susceptibility to lipid peroxidation under oxidative stress [5,6,50]. Lipid peroxidation can impair membrane fluidity, sperm motility, acrosomal function, and fertilizing capacity [5,50]. In addition, ferroptosis-related lipid oxidative injury has been increasingly discussed in male reproductive dysfunction [45]. Therefore, high-choline diet-induced reproductive phenotypes may reflect combined effects of TMAO elevation, one-carbon metabolism, phospholipid remodeling, PUFA-dependent lipid peroxidation, and ferroptosis-related vulnerability, rather than TMAO toxicity alone. Similarly, fecal microbiota transplantation or bacterial colonization experiments transfer or modify multiple microbial functions, metabolites, and immune stimuli, not only TMA production [19]. These models are useful for testing biological plausibility, but they cannot fully separate the effects of TMAO from the broader metabolic and inflammatory changes that accompany altered gut microbial function.

#### 5.3.3. Context-Dependent Effects of TMAO

TMAO should also be considered within a broader microbial metabolic network. The gut–testis axis may involve short-chain fatty acids, bile acids, indole metabolites, TMAO, and bacterial components such as lipopolysaccharide [51,52,53,54]. These signals can influence intestinal barrier function, immune responses, energy metabolism, endocrine regulation, and redox balance [9,51,52]. The butyrate-related study in asthenozoospermia provides a useful comparison: reduced butyrate-producing bacteria and lower serum butyrate were associated with impaired sperm quality in patient-derived microbiota models [55]. This does not provide direct evidence for a role of TMAO, but it shows that gut microbial metabolites may have both potentially deleterious and protective associations with spermatogenesis.

Another issue is that TMAO does not always carry the same biological meaning across contexts. Fish and seafood can provide preformed TMAO, yet fish intake is often linked to healthier dietary patterns [22,56]. TMAO may also function as an osmolyte or protein stabilizer, and its biological effects may depend on concentration, exposure duration, cell type, and tissue context [47]. Therefore, elevated plasma TMAO should not automatically be interpreted as a harmful reproductive exposure. A more balanced interpretation is that TMAO may act as a candidate metabolic signal within a wider gut microbial and host metabolic state. Future studies should evaluate TMAO together with other microbial metabolites, inflammatory markers, renal function, dietary data, and reproductive endpoints rather than treating it as an isolated causal factor [24,25,26,57].

### 5.4. Clinical Translation and Intervention Limits

The TMA/TMAO pathway is modifiable, but modifiability alone does not make it a clinical target in male infertility. Diet, gut microbiota composition, microbial TMA production, hepatic FMO3 activity, and renal clearance can all affect circulating TMAO levels [16,20,21,22,30]. This creates opportunities for intervention, but it also means that changes in TMAO do not necessarily reflect testis-specific effects. A reduction in plasma TMAO does not necessarily mean that testicular exposure, Leydig cell function, spermatogenesis, or fertility outcomes have improved.

Dietary intervention is the most straightforward example. Red meat, white meat, and non-meat protein diets differ in their effects on TMAO production and excretion [21], and plant-based diets or dietary fiber may influence TMAO production by changing substrate availability and gut microbial composition [22,58,59]. However, these studies mainly show that TMAO is diet-responsive. They do not demonstrate that changing TMAO levels improves sperm motility, total motile sperm count, testosterone levels, pregnancy rate, or live birth rate. Therefore, dietary modification should not be presented as a TMAO-targeted treatment for male infertility.

Microbiota modulation also requires caution. Probiotics, prebiotics, and synbiotics can alter gut microbial composition and may influence inflammation, intestinal barrier function, and metabolite profiles [10,54]. However, these interventions are difficult to compare because strains, doses, treatment duration, study populations, and outcome measures vary widely. The butyrate-related study in asthenozoospermia also indicates that microbiota-based strategies should not focus only on lowering TMAO, because protective microbial metabolites may also contribute to spermatogenic function [55]. A narrow TMAO-lowering approach may miss the broader metabolic balance of the gut–testis axis.

Pharmacological inhibition of microbial TMA production is even further from clinical application in male infertility. 3,3-dimethyl-1-butanol has been reported to reduce TMAO and improve atherosclerosis-related phenotypes in animal models [60], and recent reviews have discussed strategies targeting substrate supply, gut microbiota, microbial TMA production, and host FMO3-related metabolism [61]. These data are useful for understanding the pathway, but they come mainly from cardiovascular and metabolic research. They do not show reproductive benefit. FMO3-related strategies also require particular caution because FMO3 participates in the metabolism of many endogenous and exogenous compounds, and direct inhibition may lead to TMA accumulation or altered drug metabolism [16,30].

These considerations create a clear distinction between metabolic pathway modulation and reproductive clinical efficacy. In cardiovascular or metabolic research, a reduction in circulating TMAO may be interpreted as evidence that the TMA/TMAO pathway has been engaged. In andrology, however, pathway engagement is not sufficient. A lower plasma TMAO level does not establish reduced reproductive tract exposure, improved Sertoli cell support, restored Leydig cell steroidogenesis, or improved germ cell development. Therefore, TMAO-lowering should not be interpreted as clinically meaningful unless it is accompanied by reproducible improvements in male reproductive endpoints.

At present, dietary modification, microbiota modulation, and microbial TMA-lowering strategies remain biologically plausible but clinically unvalidated approaches in male infertility. Future intervention studies should determine not only whether plasma or seminal TMAO decreases, but also whether sperm motility, total motile sperm count, sperm DNA fragmentation, testosterone levels, natural pregnancy rate, assisted reproduction outcomes, or live birth rate improve. If an intervention changes TMAO but does not improve these reproductive outcomes, its value in andrology will remain limited. For this reason, the TMA/TMAO pathway should currently be viewed as a research target rather than a diagnostic or therapeutic target in male infertility.

### 5.5. Future Research Priorities

Future studies should move beyond simple associations between plasma TMAO and semen parameters. The priority is to determine whether TMAO is an independent reproductive risk signal, a marker of broader metabolic disturbance, or a downstream correlate of diet, renal function, inflammation, and gut microbial imbalance. This requires prospective human studies with repeated measurements of plasma and, where possible, seminal plasma TMAO. These studies should also include standardized semen parameters, reproductive hormones, gut microbial functional profiling, dietary records, renal function, body mass index, diabetes status, smoking, antibiotic exposure, and red meat and fish intake. Without these variables, it will remain difficult to distinguish a TMAO-specific association from general metabolic confounding.

Future work should also connect systemic measurements with reproductive tract biology. Measuring plasma TMAO alone cannot clarify whether the male reproductive tract is exposed to TMAO or whether local testicular pathways are activated. Studies should therefore assess paired plasma and seminal plasma metabolomics, including TMAO and related metabolites, together with inflammatory markers, oxidative stress markers, reproductive hormones, and gut microbial data. This approach would help determine whether circulating TMAO reflects local reproductive tract exposure or instead represents a systemic metabolic signal without direct seminal or testicular accumulation. When human testicular samples are available, validation of YAP phosphorylation, mitochondrial integrity, StAR expression, and testosterone synthesis would help determine whether the Hippo/YAP–mitochondria–StAR pathway observed in preclinical models is relevant to human testes [19]. Experimental models should also better reflect long-term human exposure rather than relying mainly on high-dose or short-term TMAO exposure.

A broader metabolic framework is also needed. Future studies should not measure TMAO in isolation. Short-chain fatty acids, bile acids, indole metabolites, and inflammatory markers such as lipopolysaccharide should be analyzed together with TMAO [24,25,26,57]. This would help determine whether TMAO is an independent risk indicator or one component of a wider gut microbial metabolic imbalance. It would also allow potentially protective and deleterious microbial metabolic branches to be evaluated within the same model.

Intervention studies should be designed around reproductive outcomes, not only metabolite reduction. Dietary changes, microbiota modulation, and pharmacological strategies may change circulating TMAO, but a lower TMAO level is not sufficient to establish clinical benefit [21,54,60,61]. Future intervention studies should assess sperm motility, total motile sperm count, sperm DNA fragmentation, testosterone levels, natural pregnancy rate, assisted reproduction outcomes, and live birth rate. Replication studies and reports of negative findings are also needed, because the current literature may overrepresent positive associations and injury-related mechanisms. Only when metabolic changes are linked to reproducible improvements in clinically meaningful reproductive endpoints can the TMA/TMAO pathway move from a research signal toward a translational target. Key unanswered questions and future research directions in TMAO-related male reproductive dysfunction are summarized in Table 3.

## 6. Conclusions

Current evidence links TMAO to asthenozoospermia and Leydig cell-related endocrine markers in humans, and to testicular injury in preclinical models. However, human studies remain observational, plasma TMAO has not been validated as a marker of reproductive tract exposure, and the Hippo/YAP–mitochondria–StAR pathway lacks independent replication or human validation. TMAO should therefore be considered as a candidate metabolic signal within the gut–testis axis, rather than as an established cause of male infertility or a therapeutic target. Prospective cohorts, multi-omics integration, human tissue validation, and intervention trials with reproductive endpoints are needed before this pathway can be considered clinically relevant in male infertility. At present, the TMA/TMAO pathway is best viewed as a research priority rather than a clinical target.

## Figures and Tables

**Table 1:** Representative human gut bacteria and associated functional genes implicated in microbial trimethylamine (TMA) production upstream of the host-derived trimethylamine N-oxide (TMAO) pathway.

Taxon or Bacterial Group	Main Pathway or Substrate	Relevance to TMA/TMAO Metabolism	Context/Functional Inference
*Phocaeicola massiliensis*	Choline-associated TMA production	Associated with the choline-to-TMA ratio and enriched in men with abnormal semen parameters [19]	Reproductive-disease-associated enrichment; functional relevance requires validation
*Veillonella* spp.	Choline-associated TMA production	Associated with the choline-to-TMA ratio and enriched in men with abnormal semen parameters [19]	Context-dependent; enrichment alone does not confirm causative TMA-producing activity
*Klebsiella pneumoniae*	Choline-associated TMA production; possible carnitine-associated TMA potential	Associated with the choline-to-TMA ratio in male reproductive metagenomic data [19]; *Klebsiella*-related *cntA* signals have been reported in human fecal TMA-producing gene analyses [29]	Strain- and context-dependent; reported both in reproductive cohorts [19] and TMA gene surveys [29]
*Clostridium* cluster XIVa strains	Choline trimethylamine-lyase gene system (*CutC*/*CutD*)	Major *cutC*-related group detected in human fecal samples [29]	Functionally linked to choline-derived TMA production; taxonomic identity alone does not predict metabolic flux
*Eubacterium* sp. strain AB3007-related taxa	Choline TMA-lyase system, *CutC*/*CutD*	Reported among human fecal *cutC*-related sequences [29]	Functionally linked to choline-derived TMA production; health relevance remains context-dependent
*Escherichia*/*Shigella*-related taxa	Carnitine oxygenase/reductase gene system (*CntA*/*CntB*)	Major *cntA*-related group detected in human fecal samples [29]	*CntA*-related TMA-producing potential; interpretation is strain-dependent
*Citrobacter* spp.	Carnitine-associated TMA production	Detected among *cntA*-related taxa [29]	Direct contribution to TMA production in the human gut requires further validation
*Desulfovibrio*-related taxa	*CutC*-related reference sequence	Reported among reference sequences linked to *cutC*-containing communities [29]	Lower-confidence taxonomic inference; human functional relevance requires further validation
*Collinsella*-related taxa	*CutC*-related reference sequence	Reported among reference sequences linked to *cutC*-containing communities [29]	Lower-confidence taxonomic inference; human functional relevance requires further validation

Note: TMA, trimethylamine; TMAO, trimethylamine N-oxide; FMO3, flavin-containing monooxygenase 3; *CutC*/*CutD*, choline trimethylamine-lyase system; *CntA*/*CntB*, carnitine oxygenase/reductase system. Gut microbiota mainly generate TMA from dietary precursors, while TMAO formation occurs mainly through host hepatic FMO3-mediated oxidation. The context/functional inference column summarizes reported enrichment or TMA-producing potential and should not be interpreted as proof of direct pathogenicity. Functional gene abundance, expression, substrate availability, microbial interactions, host FMO3 activity, and renal clearance may be more informative than taxonomic identity alone. The listed functional genes (*cutC*/*cutD* and *cntA*/*cntB*) are inferred from metagenomic, gene-targeted, or cultivation-based studies; their presence does not necessarily indicate active gene expression or metabolic flux in vivo.

**Table 2:** Summary of major studies linking TMAO to impaired spermatogenesis.

Study	Study Type and Population	Key Methods	Main Findings	Main Limitations
Xing et al., 2025 [18]	Hospital-based matched case–control study; 314 pairs of asthenozoospermia cases and normozoospermic controls	Plasma TMAO and related metabolites, including choline, betaine, and L-carnitine, were measured; conditional logistic regression was performed with semen phenotypes	The highest plasma TMAO quartile was associated with higher odds of asthenozoospermia (OR = 1.80; 95% CI: 1.16–2.81); higher choline and L-carnitine levels were associated with lower odds	Observational design; single plasma measurement; residual confounding from diet, renal function, medication use, and population source could not be fully ruled out
Ayvaci et al., 2026 [27,28]	Boys with cryptorchidism	Serum TMAO and Leydig cell-related markers, including INSL3, were measured	Serum TMAO was elevated in boys with cryptorchidism, while INSL3 levels were reduced, suggesting possible testicular endocrine involvement	Participants were not adult infertile men; gut microbiota and TMA-producing functional genes were not assessed; the source of elevated TMAO could not be determined
Wang et al., 2026 [19]	Patient microbiome analysis and preclinical mouse experiments	Fecal metagenomics; fecal microbiota transplantation (FMT); high-choline diet; TMA-producing bacterial colonization; TMAO exposure; testicular, sperm, mitochondrial, and Hippo/YAP–StAR-related assessments	Abnormal microbiota, high-choline diet, and TMAO exposure were associated with testicular injury, abnormal sperm morphology, and reduced testosterone synthesis in mice	Limited generalizability from mouse models; dose and exposure route differ from daily human exposure; lack of human testicular validation and clinically relevant reproductive endpoints
Li et al., 2017 [31]	Aged rat vascular study	TMAO, inflammation, oxidative stress, and endothelial function were measured; DMB was used to inhibit TMA production	Elevated TMAO was associated with inflammation, oxidative stress, and endothelial dysfunction; some markers improved after DMB intervention	Non-reproductive study; serves only as indirect mechanistic support and cannot directly explain testicular or sperm outcomes
Wang et al., 2019 [21]	Randomized dietary intervention study in healthy adults	Red meat, white meat, and non-meat protein diets were compared; TMAO production and excretion were assessed	A red meat diet increased systemic TMAO levels; plasma TMAO decreased within several weeks after stopping the red meat diet	Mainly demonstrates that diet and renal excretion affect TMAO levels; does not directly assess male reproductive outcomes

**Table 3:** Key unanswered questions for future studies on TMAO and male reproductive dysfunction.

Key Question	Why It Matters	Suggested Approach
Does TMAO temporally precede changes in semen quality?	Current human evidence is mainly observational and cannot establish temporal order.	Prospective cohorts with repeated plasma TMAO, semen parameters, hormone levels, and confounder assessment.
Does circulating TMAO reflect reproductive tract exposure?	Plasma TMAO may not represent seminal plasma or testicular exposure.	Paired plasma and seminal plasma metabolomics; human testicular validation when samples are available.
Is the Hippo/YAP–mitochondria–StAR pathway relevant in humans?	The current mechanism is mainly supported by preclinical evidence.	Assessment of YAP phosphorylation, mitochondrial integrity, StAR expression, and steroidogenic markers in human samples.
Is TMAO independent of broader metabolic confounding?	TMAO is influenced by diet, renal clearance, FMO3 activity, gut microbiota, and metabolic status.	Integrated models including diet, renal function, microbiome data, inflammatory markers, and microbial metabolites.
Does reducing TMAO improve reproductive outcomes?	Reducing a metabolite does not prove clinical benefit.	Intervention studies measuring sperm quality, testosterone, pregnancy rate, assisted reproduction outcomes, and live birth rate.

## Data Availability

No new data were created or analyzed in this review.

## Abbreviations

The following abbreviations are used in this manuscript:

ATP	Adenosine triphosphate
BMI	Body mass index
CI	Confidence interval
CKD	Chronic kidney disease
*CutC*/*D*	Choline trimethylamine-lyase system
DMB	3,3-Dimethyl-1-butanol
DNA	Deoxyribonucleic acid
FMO3	Flavin-containing monooxygenase 3
FMT	Fecal microbiota transplantation
INSL3	Insulin-like peptide 3
LH	Luteinizing hormone
LPS	Lipopolysaccharide
NLRP3	NLR family pyrin domain containing 3
OR	Odds ratio
PRISMA	Preferred Reporting Items for Systematic Reviews and Meta-Analyses
ROS	Reactive oxygen species
StAR	Steroidogenic acute regulatory protein
TMA	Trimethylamine
TMAO	Trimethylamine N-oxide
YAP	Yes-associated protein
YAP1	Yes-associated protein 1
*cntA*/*cntB*	Carnitine oxygenase/reductase gene system
OXPHOS	oxidative phosphorylation
OPA1	optic atrophy 1
DRP1	dynamin-related protein 1
CHOP	C/EBP homologous protein
eIF2α	Eukaryotic initiation factor 2 alpha
ER	Endoplasmic reticulum
PERK	Protein kinase RNA-like endoplasmic reticulum kinase
UPR	Unfolded protein response
BTB	Blood–testis barrier
PUFAs	Polyunsaturated fatty acids
